# Factors impacting HIV testing among young sexually active women migrant workers in Vietnamese industrial zones

**DOI:** 10.1186/s12889-023-16841-y

**Published:** 2023-10-06

**Authors:** Toan Ha, Hui Shi, David Givens, Trang Nguyen, Nam Nguyen

**Affiliations:** 1 Department of Infectious Diseases and Microbiology, School of Public Health, University of Pittsburgh, Pittsburgh, PA USA; 2 Department of Epidemiology, School of Public Health, University of Pittsburgh, Pittsburgh, PA USA; 3 Institute of Social and Medical Studies, Hanoi, Vietnam

**Keywords:** HIV testing, Women migrant workers, Industrial zones, Vietnam

## Abstract

**Background:**

Young migrant workers living in low- and middle-income countries often experience barriers and inadequate access to HIV prevention and treatment services. This study examines the prevalence of HIV testing, associated factors, and reasons for obtaining and not obtaining HIV testing among young sexually active women migrant workers in an industrial zone in Hanoi, Vietnam.

**Methods:**

A cross-sectional study was conducted among 512 sexually active young women migrant workers (aged 18 to 29) working in the Thang Long industrial zone in Hanoi, Vietnam. Data was collected via a face-to-face interview from January 2020 to June 2021. Multivariable logistic regression analysis was used to explore factors associated with ever-testing for HIV among sexually active participants.

**Results:**

The study found a low level of HIV testing and high rates of unprotected sex. Among those who reported being sexually active, only 23.7% of participants (*n* = 126) reported having ever been tested for HIV. Among those who reported never having tested for HIV, 38.2% reported not using condoms during their most recent sexual encounter. Factors associated with engaging in HIV testing included being older (25–29 years), having greater knowledge about HIV, past use of sexual and reproductive health and HIV services, and familiarity with HIV testing locations.

**Conclusions:**

Overall, a low level of HIV testing, high rates of unprotected sex, and low perceived risks regarding HIV among the study participants point to a need to implement targeted HIV interventions that can improve both safe sex practices and perceptions of and knowledge about risky sexual behaviors. Such interventions should use insights from this study to address factors facilitating HIV testing among industrial zones’ women migrant workers.

**Supplementary Information:**

The online version contains supplementary material available at 10.1186/s12889-023-16841-y.

## Background

Despite remarkable global progress in HIV prevention and treatment, HIV remains a major public health challenge throughout the world. Globally, in 2020 alone an estimated 1.5 million people newly acquired HIV, raising the current estimated number of people living with HIV (PLWH) to 37.7 million [1]. Furthermore, as of 2020 approximately 6.1 million PLWH are unaware of their positive status. Worldwide, the vast majority of PLWH live in low- and middle-income countries; the Asian-Pacific region has the second largest population of PLWH of any world region after sub-Saharan Africa [1].

In recent decades, low- and middle-income countries have experienced increasing internal rural to urban economic migration as economic growth and globalization spur rural workers to seek better livelihoods in cities [2]. While rural to urban migration frequently improves workers’ economic situations, work in urban areas increases sexual health vulnerabilities, including HIV infections [3]. Improving HIV testing uptake among high-risk populations, including migrant workers, is one of the priorities of the UNAIDS 95/95/95 targets [4]. These targets, established by UNAIDS in 2020 and endorsed by United Nations member states in June 2021, seek to eradicate the HIV epidemic by 2030. They aim to ensure that 95% of PLWH know their status, 95% of diagnosed individuals receive antiretroviral therapy, and 95% of those on treatment achieve suppressed viral loads.

However, existing evidence and epidemiological data within the Asian-Pacific region show that those who migrate experience higher HIV prevalence than the general populations where they live [5]. While migrants do account for a large number of HIV infections worldwide [6] and have higher HIV prevalence than the general population [5], it is also well documented that people who migrate are a key demographic group experiencing high risks and vulnerabilities related to HIV acquisition [7–10]. For instance, a systematic review of studies among rural to urban migrants in China found that HIV prevalence among women migrants is 12 times higher than in the general population [11]. In Thailand, HIV prevalence among migrants from Cambodia, Myanmar, and Vietnam is nearly four times the HIV prevalence among the general population [7]. In Africa, a study in Lesotho documented that HIV prevalence among textile factory migrant workers was higher than other adults (42.7% vs 25%) [12]. Within Vietnam, new research is now beginning to shed light on the conditions and resources of migrant workers, including the prevalence of unsafe sexual behaviors and limited knowledge about sexually transmitted infections and HIV acquisition [13, 14].

Indeed, migrant workers’ vulnerabilities are known to often stem from barriers related to education [15], separation from family or family instability, new and inequitable urban behavioral resources and expectations [2], limited knowledge of HIV, limited access to health care and testing sites [16], language barriers [17], and exploitative and unstable working conditions, and sexual violence [2, 18]. Migrants’ increased HIV vulnerabilities are further compounded by a frequent lack of, or inadequate access to, HIV prevention, treatment, and support services [19]. A 2013 systematic review by Alvarez-del Arco et al.summarized migrants’ barriers to HIV testing as spanning multiple forms and iterations of administrative, legal, and cultural barriers [5] as well as long travel times to and extended waiting times at testing centers [20]. Furthermore, a recent study in China have found that transnational migrants in China had low HIV testing uptake because of a perceived low need for testing, unwillingness to test and time constraints [21]. HIV-related stigma in many Asian countries, including both Vietnam and China, continues to function as a uniquely insidious and pervasive barrier as well [22–25].

Among Asian-Pacific low- and middle-income countries, Vietnam possesses unique socio-economic structures relevant to HIV transmission. Rural-to-urban migration is rising in Vietnam as the country has experienced rapid growth and rapid industrialization over the past two decades. Since Vietnam adopted a liberalized economic model in 1986, the country has attracted large numbers of foreign investors opening factories in industrial zones (IZ). The country currently has 257 operational IZs and approximately 3.6 million workers; the great majority of them are women from rural areas [26]. These migrant workers predominantly come from low income, patriarchal families in which they have typically had limited formal education, opportunities, and mobility [27, 28]. These women migrant workers in the IZs are often particularly vulnerable to HIV acquisition as male supervisors, male peers at work, male landlords and shopkeepers, and men in the community may seek their disposable income and perceived vulnerability and/or sexual availability. These risks occur in a societal context of gender inequality, limited social supports, and patriarchal cultural norms which constrains young women’s capability to negotiate safer sex and control their own sexual activity and risk of exposure to HIV [29]. Indeed, previous studies have identified that factors impacting uptake of HIV testing among women migrant workers include levels of education generally and HIV/AIDS knowledge specifically, perceptions of HIV risk and knowledge of HIV testing locations [30], access to a medical check-up within the past 12 months, having an employment contract, being sexually active in the past six months [31], or having had a previous STI diagnosis [32].

Despite a decrease in overall HIV prevalence in Vietnam (0.3% of the population aged 14 – 49 in 2022), HIV remains a serious public health concern, with an estimated 5,200 new HIV infections and 5,000 AIDS-related deaths in 2020 [33]. Today, Vietnam is home to an estimated 250,000 PLWH, with higher incidence rates in key populations including sex workers, men who have sex with men, and intravenous drug users. However, an increase in HIV infections among IZ workers has also recently been documented [34]. Although migrants are one of the priority groups for HIV prevention programs in Vietnam’s National Strategy to end the AIDS epidemic by 2030 [35], a lack of both specific policies and surveillance and behavioral data on migrant workers perpetuate barriers to effective HIV prevention interventions among migrant workers in general and IZ migrant workers in particular [36]. While HIV testing is free and widely available across the country, migrant workers still face multiple barriers that impede their uptake of HIV testing as described above [28, 29]. Further, a 2015 report from the Vietnam Ministry of Health indicated that reproductive health services in the country are not responsive to the specific needs of vulnerable groups, including migrants and PLWH [37].

In summary, migrant women workers in Vietnam comprise a vulnerable demographic group impacted by HIV. This problem is compounded by the paucity of information about HIV testing behavior and factors impacting testing uptake among sexually active women migrant workers living and working in Vietnam’s industrial zones. The purpose of this study is to examine the prevalence of HIV testing and associated factors impacting testing among IZ sexually active women migrant workers. The results of these two goals are crucial for informing the development and implementation of effectively tailored HIV interventions to prevent HIV and increase HIV testing among IZ sexually active women migrant workers.

## Methods

### Study site and population

This study used data that was part of the study project “HIV risk among young women migrant workers in the industrial zones in Vietnam” which is described in detail in prior publications [38, 39]. In brief, a cross-sectional study was conducted among young migrant women workers in Thang Long Industrial Park on the outskirts of Hanoi, the capital of Vietnam from January 2019 to November 2020. Eligibility criteria for participation in the study included: (1) self-identifying as a woman; (2) being between 18 and 29 years of age; (3) either single, currently married but not living with a husband or partner while working in the IZ, separated, divorced, or widowed; (4) having worked in the IZ for six or more months; (5) from a rural area or another province prior to IZ employment. The study specifically focused on recruiting individuals who are either single or currently married but not cohabiting with a husband or partner while working in the industrial zone. This focus was driven by the original hypothesis of the study that individuals who are currently married but not living with a husband or partner might face a heightened risk of HIV due to potential extramarital relationships.

### Procedures

A cluster sampling method was used to select participants. Cluster sampling frequently results in intra-class correlation, where participants within the same cluster tend to report similar outcomes. This correlation could potentially affect outcome independence in statistical tests. To address this concern, we used the method proposed by Killip et al. [40] to adjust the impact of intra-class correlation when calculation of the study power and sample size. The study sampling method and sample size was described in detail in a prior publication [39]. In summary, a total of 779 rent clusters and two dormitories located near the industrial zone were mapped and enumerated. After selecting rent clusters with at least six eligible participants, 360 clusters were excluded, resulting in 419 qualifying clusters. Out of the 1316 eligible workers contacted from 419 qualifying rent clusters, only 936 participants agreed and consented to participate in the study. From the two dormitories, 125 out of 175 eligible participants agreed to join the study, resulting in a total of 1061 participants participating in the study. Of these participants, 531 individuals reported a history of sexual activity, thus forming the final sample for this study. Eligible participants who agreed to participate in the study were fully informed about the study and signed the conformed. Then, they participated in a face-to-face hour-long interview using closed-ended questions conducted by trained field researchers.

The study was approved by the University of Connecticut Health Center Institutional Review Board and the Institute for Social and Medical Studies, Vietnam (IRB Number: 19-134O-1). Written informed consent in Vietnamese was obtained from all participants in the study.

### Measures

#### Demographics

The study collected demographic characteristics of participants including age, marital status, education, ethnicity, residence and monthly income.

*HIV lifetime testing* was evaluated by asking participants if they knew where to get an HIV test and if they had ever been tested, including reasons for testing decisions.

*Sexual behavior and condom use* was assessed by asking participants about their sexual history and condom usage, focusing on sexual activities within the past six months and condom use at the last sexual encounter.

#### HIV/AIDS knowledge

Knowledge of HIV/AIDS was measured with seven “yes”, “no”, “do not know” questions that had been previously vetted and used among young people in Vietnam [41, 42]. Participants were asked questions about HIV transmission, HIV prevention and common HIV misconceptions (e.g., People can protect themselves from HIV infection by using a condom correctly every time they have sex). Each correct answer was given one point, with a total score ranging from zero to seven points. A higher score indicated greater HIV knowledge.

#### Perceived risk of HIV

Perceived risk of HIV infection was measured using two question assessing participants’ perception about the possibility that they and their sexual partner (for those who had one or more) would acquire HIV. The response format was a five-point scale ranging from extremely unlikely (1) to extremely likely (5).

#### Utilization of sexual and reproductive health and HIV (SRH/HIV) services

We asked participants if they had used SRH or HIV services since they had begun working in the IZ.

### Statistical analysis

ANOVA and chi-square tests were employed to compare the distributions of continuous and categorical variables. Logistic regression models were conducted to explore factors associated with HIV testing among sexually active migrant women. First, we fit univariate logistic regression models to examine the association between HIV testing and each sociodemographic characteristic, condom use during most the recent sexual encounter, HIV knowledge, perceived HIV risk, use of SRH/HIV services and familiarity with HIV testing site. Only those variables that were significant at *p* < 0.25 in the bivariate models were included in the multivariable regression models. *P*-value < 0.05 was considered statistically significant. All analyses were performed using Stata 17.0 (Stata Corp, College Station, TX).

## Results

### Sample characteristics

Study participants were on average 24.8 years of age (ranging: 19 to 29, standard deviation = 3.1). Of the total 531 participants, 41.9% were single. The majority of respondents had completed high school (10–12 years of education). Nearly 86% of participants were living in rent clusters. While most of the respondents worked 8 h or less a day, nearly 24% were working over 8 h a day. Mean monthly income was US$297 per month, ranging from $195 to $652 (Table 1).

**Table 1:** Characteristics of study participants by ever-testing for HIV among sexually active migrant women

**Characteristics**	**All**	**Ever having taking HIV test**		***P*-value**
	***n* = 531 (100%)**	**No,** ***n* = 405 (76.3%)**	**Yes,** ***n* = 126 (23.7%)**	
**Age(years), n (%)**				** < 0.023**
18–24	249 (46.9%)	201 (49.6%)	48 (38.1%)	
25–29	282 (53.1%)	204 (50.4%)	78 (61.9%)	
**Marital status, n (%)**				< 0.444
Unmarried	215 (41.9%)	167 (42.9%)	48 (59.7%)	
Married	297 (58.1%)	222 (57.1%)	75 (40.3%)	
**Ethnicity, n (%)**				0.159
Kinh (the majority)	343 (64.6%)	88 (69.8%)	133 (62.9%)	
Other minority groups	188 (35.4%)	38 (30.2%)	53 (37.1%)	
**Education background, n (%)**				**0.020**
5–9 years	84 (10.3%)	66 (9.7%)	18 (12.9%)	
10–12 years	379(79.3%)	287 (80.9%)	92 (72.0%)	
$\geq12\;\mathrm{years}$	68 (10.4%)	52 (9.4%)	16 (15.1%)	
**Monthly income (USD), mean (SD)**	531 (56.5)	294.9 (59.0)	302.0 (65.1)	0.251
**Residence type**				0.670
Dormitory	75 (11.8%)	60 (14.8%)	15 (11.9%)	
Rent cluster	456 (88.2%)	345 (85.1%)	111 (88.1%)	
**Using a condom during last sexual encounter, n (%)**				0.148
No	211 (39.7%)	154 (38.1%)	57 (45.2%)	
Yes	320 (60.2%)	251 (61.9%)	69 (54.8%)	
**Knowledge of HIV/AIDS, mean (SD)**	5.5 (1.0)	5.5 (1.0)	5.7 (1.0)	**0.027**
**Knowing one HIV Testing Site**				** < 0.001**
No	133 (23.0%)	124 (30.6%)	9 (7.1%)	
Yes	398 (77.0%)	281 (69.4%)	117 (92.9%)	

*Abbreviation: SRH/HIV*: Sexual and Reproductive Health/HIV

### HIV testing behaviors

#### Reasons for HIV testing

Among our eligible population (participants who reported being sexually active), less than twenty-four percent (23.7%) reported having undergone HIV testing at some point (Table 2). The primary reasons for getting tested were as follows: 34.7% mentioned that HIV screening was included in their company’s routine health check-ups, 27.1% had received a recommendation from a physician, 10.3% expressed concern about potential HIV exposure, and 7.5% reported undergoing testing as part of antenatal care. Notably, those who received a recommendation from a physician were more likely to undergo the test (*p* = 0.012). Additional motivations for taking the test included participating in free testing initiatives led by NGO projects, donating blood, and a general desire to ensure their personal safety.

**Table 2:** Reasons for HIV testing

**Characteristics**	**All**	**Marital status, n (%)**		***P-Value***
	***n* = 123** **(100%)**	**Unmarried** ***n* = 48 (39.1%)**	**Married** ***n* = 75 (60.9%)**	
**Healthcare providers recommendations**				**0.012**
No	89 (72.9%)	35 (72.9%)	54 (72.0%)	
Yes	34 (27.1%)	13 (27.1%)	21 (28.0%)	
**Worry about being exposed to HIV**				0.940
No	113 (89.7%)	42 (87.5%)	68 (90.3%)	
Yes	13 (10.3%)	6 (12.5%)	7 (9.3%)	
**Routine health checkup**				0.091
No	73 (59.3%)	24 (50.0%)	49 (65.3%)	
Yes	50 (40.6%)	24 (50.0%)	26 (34.7%)	
**Antenatal care checkup**				** < 0.002**
No	110 (92.5%)	48 (100%)	62 (82.7%)	
Yes	13 (7.5%)	0 (0.0%)	13 (17.3%)	
**Other reasons**^a^				0.300
No	109 (86.6%)	99 (89.2%)	63 (84.0%)	
Yes	17 (13.4%)	12 (10.8%)	12 (16.0%)	

^a^Other reasons included: a free testing offered by an NGO project, requirements for blood driving; wanting to be safe

#### Reasons for not receiving HIV testing

The most common reason for having never been tested for HIV was a perceived low risk for HIV exposure (93.1%), followed by structural barriers (e.g., not knowing where to go for a test, concern about the cost, etc.) (4.1%) and lack of time to pursue testing (3.1%) (Table 3). Other reasons (cumulatively 3.4%) included not having received a physician’s recommendation, fear of testing positive, fear of being judged, and loss of confidentiality. Of those who reported structural barriers as their main reason for not testing, 78.2% and 21.7% reported not knowing where to go for a test or concern about the cost, respectively (data not shown).

**Table 3:** Reasons for not testing for HIV

**Characteristics**	**All**	**Marital status, n (%)**		***P-value***
	***n* = 388 (100%)**	**Unmarried** ***n* = 166 (42.9%)**	**Married** ***n* = 222 (57.1%)**	
**Low perceived risk for HIV**				0.532
No	27 (6.9%)	10 (6.1%)	17 (7.7%)	
Yes	361 (93.1%)	156 (93.9%)	205 (92.3%)	
**Structural barriers**^a^				0.6638
No	372 (98.9%)	160 (96.4%)	212 (95.5%)	
Yes	16 (4.1%)	6 (3.6%)	10 (4.5%)	
**Fear of judgment or being embarrassed**				0.719
No	382 (98.2%)	163 (98.2%)	219 (98.5%)	
Yes	6 (1.8%)	3 (1.8%)	3 (1.5%)	
**Lack of time**				0.502
No	376 (96.9%)	162 (97.6%)	214 (96.4%)	
Yes	12 (3.1%)	4 (2.4%)	8 (3.6%)	
**Other reasons**^b^				0.401
No	381 (96.6%)	160 (96.1%)	221 (97.8%)	
Yes	10 (3.4%)	6 (3.9%)	3 (2.2%)	

^a^ Structural barriers included: did not know where to go for a test or worried about the cost

^b^ Other reasons included: not having received physician recommendation, fear of testing positive, fear of being judged, and loss of confidentiality

### Univariable and multivariable analysis of factors associated with HIV testing

In univariate logistic regression analyses, older age (OR = 1.60; 95% CI: 1.06, 2.41), greater HIV/ADIS knowledge (OR = 1.25; 95% CI: 1.03, 1.53), familiarity with HIV testing site (OR = 5.73; 95% CI: 2.82, 11.67) and usage of SRH/HIV services while living in the industrial zone (OR = 1.57; 95% CI: 1.08, 1.17) were significantly associated with an increased odds of previous HIV testing. Results from multivariable logistic regression analysis indicated that participants had 38% higher odds of testing for every one-point increase in their HIV knowledge (aOR = 1.38; 95% CI = 1.16, 1.63) (Table 4). Participants familiar with an HIV testing location were over seven times more likely to report HIV testing (aOR = 7.66; 95% CI = 1.16, 1.63). Finally, the odds of HIV testing among those who utilized sexual and reproductive health/HIV services during their tenure in the industrial zone were 1.36 times higher compared to those who did not use such services (aOR = 1.36; 95% CI = 1.11, 2.21).

**Table 4:** Factors associated with ever-testing for HIV among sexually active migrant workers (*n* = 531)

**Characteristics**	**OR (95% CI)**	***P- value***	**aOR (95% CI)**	***P-value***
**Age (years)**				
18–24	Ref		Ref	
25–29	1.60 (1.06–2.41)	**0.024**	1.32 (0.86—2.13)	0.188
**Marital status, n (%)**				
Unmarried	Ref			
Married	1.17 (0.78–1.77)	0.444	NI	
**Ethnicity**				
Other minority groups	Ref		Ref	
Kinh	1.37 (0.89–2.09)	0.159	1.32 (0.84 – 2.08)	0.226
**Education background**				
5–9 years	Ref			
10–12 years	1.17 (0.66–2.08)	0.580	NI	
$\geq12\;\mathrm{years}$	1.12 (0.52–2.42)	0.757	NI	
**Monthly income (USD)**	1.00 (0.99–1.01)	0.240	1.00 (0.99—1.00)	0.448
**Residence type**				
Dormitory	Ref			
Rent cluster	1.28 (0.70–2.35)	0.414	NI	
**Sexual behavior and condom use at last sexual encounter**				
Having sex without condoms	Ref		Ref	
Having sex with condoms	0.74 (0.49–1.11)	0.149	0.75 (0.48 – 1.17)	0.216
**Knowledge of HIV/AIDS, mean**	1.25 (1.03–1.53)	**0.027**	1.27 (1.02—1.58)	**0.035**
**Perceived HIV risk**				
Low	Ref			
Median	0.78 (0.51–1.19)	0.252	NI	
High	1.07 (0.46–2.57)	0.873	NI	
**Knowing HIV testing site**				
No	Ref		Ref	
Yes	5.73 (2.82–11.67)	** < 0.001**	6.77 (3.17–14.44)	** < 0.001**
**Used SRH/HIV services while living in the IZ**				
No	Ref		Ref	
Yes	1.57 (1.05–2.35)	**0.030**	1.36 (1.11–2.57)	**0.046**

*aOR*: adjusted Odd Ratio, *CI*: Confidence interval, *NI*: Not included in multivariable modeling because not significant in univariate logistic regression analysis

## Discussion

This study documented that a low proportion (23.7%) of sexually active migrant women workers reported having ever tested for HIV. By comparison, the level of HIV testing among this population was similar to that of studies among internal women migrant workers in China (23%) [32] and lower than the of studies among internal women migrant workers in India (43.3%) [43]. Of particular concern is that among those who had never tested for HIV, 38% of those who reported having sex did not use a condom during their last sexual encounter. A low level of HIV testing, combined with a high level of sexual activity without condoms, leaves women migrant workers at high risk of HIV and other STIs. These findings demonstrate that HIV/STI interventions are highly indicated for this underserved, high-risk population.

Another key finding is that low perceived risk of HIV is a major reason for participants not testing, which is similar to results in other recent study in China [21]. Of the women migrant workers who reported having low perceived risk for HIV (*n* = 123), 58.9% of them did not use condoms during their last sexual encounter. This finding supports previous work which found a low rate of condom use during sex among factory workers in Vietnam [13]; however, our current study identifies a direct disconnect between objectively risky sexual behavior and perceptions of risk. In other words, it is clear that participants’ perception of low HIV risk was not supported by their reported risky sexual behaviors. This misperception suggests a need for educational interventions to improve participants’ understanding of the severity of risky sexual behaviors, to close the gap between perceived and real risk of HIV.

Concerning the social barriers and gendered expectations associated with researching sexual practices, a previous study by Weinstein and Nicolich (1993) documented that people may continue or be compelled to continue engaging in risky sexual behaviors even after they acknowledge the dangers or personal ramifications posed by HIV acquisition [44]. These individuals may have wanted to adopt preventive behaviors but felt unable to do so due to their partners dismissal of safer sex practices. Such findings highlight some of the gendered social norms that may make it difficult for women and all gender minorities to negotiate safer sex in many settings [45]. As this relates to our study, young women migrant workers may not always have sufficient interpersonal power to make/enact safe decisions about condom use when having sex. Traditional patriarchal cultural norms in Vietnam, which typically include the cultural expectation of women’s submission in sexual activities, still frequently constrain young women’s capability to negotiate safer sex and control their own sexual activity. Such gender-based assumptions leave many people, including those who identify as women, vulnerable to HIV and numerous other sexual health risks [31]. This insight may hold implications for possible interventions. Specifically, engaging men and addressing gender inequality in HIV programs could be an important strategy to reduce overall HIV risks, which would then also benefit IZ women migrant workers [46].

Consistent with previous studies among migrants in Thailand [47] and other countries [48], our findings showed that women migrant workers who possessed higher HIV knowledge were more likely to report having ever tested for HIV. Alongside similar findings from scholars in other countries, our findings suggest that improving HIV knowledge among migrant workers in Vietnamese IZ settings may help improve HIV testing behaviors among this population. Indeed, a systematic review in low- and middle-income countries in 2020 found that education interventions helped improve both HIV knowledge and the uptake of HIV testing factory workers [49]. Given the increasing use of smartphones among young people—including migrant workers in Vietnam [50]-interventions using mobile applications such as mHealth (a mobile application for HIV prevention) may provide an effective approach to improve HIV knowledge and HIV testing among this population. For example, the use of mHealth has been shown to be effective at increasing HIV knowledge and uptake of HIV testing among young Indonesian key populations [51], as well as increasing women migrant workers’ general sexual and reproductive health knowledge in Vietnam [52].

Finally, the data from this study demonstrate that women migrant workers who had used SRH/HIV services while living in the IZ were over two times more likely to report having tested for HIV. In addition, the most common reasons for HIV testing were routine occupational health checkups (43.0%) and receiving healthcare providers recommendations (31.2%). This finding suggests that access to general healthcare services for migrant women workers increases their HIV testing rate, and so improving access to these services would further bolster HIV testing.

### Limitations

Our study has some limitations. The cross-sectional nature of the study design, for example, does not allow for causal inferences. Our study participants were recruited from only one IZ; while the Thang Long Industrial Park is demographically similar to many other IZs around Hanoi, it is not necessarily representative of all IZs in Vietnam. Therefore, generalizations of the findings to other women migrant workers in other locations should be made with caution. HIV testing was self-reported and was not confirmed by medical records; thus, it is possible that recall bias and/or social desirability biases may have occurred during self-reporting. Additionally, gender does not exist in a binary, and this study intentionally uses the broad and non-biologically deterministic term ‘women’ throughout to include all participants who identify as women regardless of their sex assigned at birth. Further research is warranted for understanding the experiences of individuals who identify as transwomen, transgender individuals, or gender nonbinary folk, as their HIV testing and other SRH experiences may well differ from the results reported here. Another limitation is our focus on self-reporting sexual activity. Our sample is dependent on accurate self-representation around sexual activity, which may be influenced by factors such as perceived social desirability bias or the experience of nonconsensual sex acts (i.e., victims may implicitly define “sexual activity” as only applying to consensual sex only) [53]. Finally, it is important to note that the study includes participants who reported having “ever tested” for HIV, and this response might include/reference time or experiences before participants were working in the industrial zone. Nevertheless, we feel that these limitations are generally outweighed by the study’s strengths, including a large sample size and novel subject; it is one of only a few studies exploring factors impacting HIV testing among an under-resourced and at-risk demographic, and is the only known study of its kind to do so specifically in Vietnam.

## Conclusion

Understanding the barriers and characteristics impacting HIV testing among high-risk migrant populations in low- and middle-income countries is critical to continuing the fight against HIV. This study adds to that important and understudied goal in Vietnam through the examination of HIV testing characteristics among a vulnerable population: young women migrant workers in IZs. Recruiting from an Industrial Park outside Hanoi, this study found low levels of HIV testing and high rates of unprotected sex among our study participants. These findings indicate an urgent need to provide or improve culturally sensitive educational materials and interventions that can impact safe sex knowledge and practices, perceptions of risky sexual behaviors, and general HIV knowledge. Furthermore, our findings suggest that improving access to healthcare resources generally, and SRH/HIV services specifically, would improve HIV testing rates among this population.

### Supplementary Information

**Additional file 1.**

## Data Availability

The data presented in this study are available on request from the corresponding author.

## Abbreviations

PLWH: People living with HIV
LMICs: Low- and middle-income countries
IZ: Industrial zones
