# Supplementary material for: Factors impacting HIV testing among young sexually active women migrant workers in Vietnamese industrial zones
Source: BMC Public Health. 2023 Oct 6;23:1938. doi: 10.1186/s12889-023-16841-y (PMC10559500; doi:10.1186/s12889-023-16841-y)
Supplement: Supplementary file 1 — Additional file 1. [file 12889_2023_16841_MOESM1_ESM.docx]

| **Title:** “**HIV risk among young women workers in the industrial zone in Vietnam**”  **Survey Questionnaire**  Contents  [SECTION 1: SOCIODEMOGRAPHIC INFORMATION 4](#_Toc145595373)  [SECTION 2: KNOWLEDGE ABOUT HIV/AIDS 6](#_Toc145595374)  [SECTION 3: PERCEIVED RISK ABOUT HIV 7](#_Toc145595375)  [SECTION 4: HIV TESTING 8](#_Toc145595376)  [SECTION 5: HIV PREVENTION 9](#_Toc145595377)  [SEXUAL BEHAVIORS - SELF-ADMINISTRATION 10](#_Toc145595378)  [HOW TO FILL THE ANSWER: 10](#_Toc145595379)  [SECTION 6: SEXUAL BEHAVIORS 10](#_Toc145595380)  [SECTION 7: USE OF HEALTH SERVICES 13](#_Toc145595381) | | | | |
| --- | --- | --- | --- | --- |
| **SECTION 1A: IDENTIFICATION** | | | | |
|  | Date of interview  (DD/MM/YYYY) | _____/_____/2020 | | |
|  | Participant ID (Participant unique code) |  | | |
|  | Date of consent taken  (DD/MM/YYYY) | _____/_____/2020 | | |
|  | Interviewer code |  | | |
|  | Start time  (HH/MM) | _____/_____ | | |
|  | End time  (HH/MM) | _____/_____ | | |
|  | Location of interview | At rent room 1  Rent cluster 2  Room in the dormitory 3  Dormitory 4  Commune health center 5  Other (specify) 6 | | |
| **SCREENER (ELIGIBILITY CRITERIA)** | | | | |
|  |  | **Yes** | **No** | |
|  | Over 18 – 24 | 1 | 2 | |
|  | From a other province/district | 1 | 2 | |
|  | In the IZ for at least 6 months (less < 5 years) | 1 | 2 | |
|  | Single or married and not living with husband | 1 | 2 | |
|  | Live in rent cluster or dormitory | 1 | 2 | |

| SECTION 1: SOCIODEMOGRAPHIC INFORMATION *Thank you for agreeing to participate in this study. First, I will be asking you some general questions about your age, where you live, your marital status, and your work.*  **[INTERVIEWER: CIRCLE ONE BEST ANSWER FOR EACH QUESTION UNLESS OTHERWISE INSTRUCTED]** | | | | | | |  |
| --- | --- | --- | --- | --- | --- | --- | --- |
| **QID** | **Question** | | **Answers** | | **Skip** | |  |
|  | | In what month and year were you born? | | Month  Year  Do not know month and/or year 99 | |  | |
|  | | What is your marital status? | | Single 1  Married and living with husband 2  Married and not living with husband 3  Not married and living with a partner 4  Widowed, divorced or separated 5  Refuse 9 | |  | |
|  | | What is the highest level of school you completed? | | No school-Illiterate 1  Primary 2  Secondary 3  High school 4  Vocational school 5  College or higher 6 | |  | |
|  | | What is your ethnicity? | | Kinh 1  Other (specify) 6 | |  | |
|  | | How long have you worked in the IZ?  *(Less than 1 year, record number of month, more than 1 year, record number of years with decimel)* | | Months  Years **.** | |  | |
|  | | How many days do you work a week? | | days | |  | |
|  | | When is your shift now?  **(Multiple choices)** | | Morning 1  Afternoon 2  Evening 3  Night 4 | |  | |
|  | | How many hours is your daily shift? | | hours | |  | |
|  | | What kind of job do you do in the IZ? | | Installation of electronic parts 1  Making garments 2  Medical equipment manufacture 3  Packaging 4  Other (specify) 6 | |  | |
|  | | On average, how much income do you earn per month in the IZ? | | VND | |  | |
|  | | Do you work extra time? | | Yes 1  No 2 | |  | |
|  | | Do you have other sources of income? | | Yes 1  No 2 | | **2 🡪Q114** | |
|  | | If yes, how much do you earn from those other sources of income per month? | | VND | |  | |
|  | | Do you have health insurance? | | Yes, I have a company's health insurance 1  Yes, I have health insurance but not from  company 2  No, I have no health insurance 3 | |  | |

| SECTION 2: KNOWLEDGE ABOUT HIV/AIDS/STIs *I would like to ask you questions about your understanding on HIV/AIDS and STIs. This is for research purpose, so there will be no right or wrong answers. You just provide me your answers based on your best knowledge.* | | | | | | |
| --- | --- | --- | --- | --- | --- | --- |
| 201 | Have you ever heard of HIV or a disease called AIDS? | Yes 1  No 2  Don’t know 9 | | | **2 or 9 skip to Q301** | |
| 202 | How can HIV be transmitted?  **(Multiple choices)** | Unprotected sexual intercourse 1  Mother-to-child transmission 2  Sharing needle 3  Unsafe blood transfusion 4  Don’t know_________________9 | | |  | |
|  | I will read each statement, please answer “Yes” or “No” for each  ***(Interviewer reads each statement)*** | | **Yes** | **No** | | **Dont’ know** |
| 203 | Can people protect themselves from HIV infection by having one uninfected faithful sex partner? | | 1 | 2 | | 9 |
| 204 | Can people protect themselves from HIV infection by using a condom correctly everytime they have sex? | | 1 | 2 | | 9 |
| 205 | Can people protect themselves from HIV by abstaining from sexual intercourse | | 1 | 2 | | 9 |
| 206 | Can a person get HIV from a mosquito bite? | | 1 | 2 | | 9 |
| 207 | Can a person get HIV by sharing food with someone who is infected? | | 1 | 2 | | 9 |
| 208 | Can a healthy-looking person have HIV? | | 1 | 2 | | 9 |

| SECTION 3: PERCEIVED RISK ABOUT HIV | | | |
| --- | --- | --- | --- |
| **QID** | **Question** | **Answers** | **Skip** |
| 301 | How likely do you think it is that you could contract HIV?  **(Show card)** | Extremely unlikely 1  Very unlikely 2  Somewhat likely 3  Very likely 4  Extremely likely 5 |  |
| 302 | **Ask if Q301=1**  How likely is it that your sexual partner (boyfriend, husband or other partner) contract HIV?  **(Show card)** | Extremely unlikely 1  Very unlikely 2  Somewhat likely 3  Very likely 4  Extremely likely 5 |  |

| SECTION 4: HIV TESTING | | | |
| --- | --- | --- | --- |
| **QID** | **Question** | **Answers** | **Skip** |
| 401 | Do you know a place where you can get an HIV test | Yes 1  No 2 |  |
| 402 | Have you ever taken an HIV test? | Yes 1  No 2 | 2🡪 Q404 |
| 403 | If yes, do you know your HIV serostatus? | ­Yes 1  No 2 | 2🡪 Go to Q406 |
| 404 | If no, have you ever thought of getting an HIV test | Yes 1  No 2 |  |
| 405 | What are reason(s) preventing you from getting HIV testing  **(Multiple choices)** | Don't believe at risk 1  Doctor never suggested 2  Don't know where to go 3  Felt judged or embarrassed 4  Worried about cost 5  Worried about confidentiality 6  Worried about results 7  Other (specify) 8 |  |
| 406 | What are reason(s) that motivated you to get HIV testing  **(Multiple choices)** | Health care provider suggested 1  Concerned might have been exposed 2  Husband/partner suggested 3  Friends suggested 4  Other (specify) 6 |  |

| SECTION 5: HIV PREVENTION | | | |
| --- | --- | --- | --- |
| **QID** | **Question** | **Answers** | **Skip to** |
| 501 | How effective are condoms at preventing HIV?  **(Show card)** | Very effective 1  Somewhat effective 2  Not too effective 3  Not at all effective 4 |  |
| 502 | How effective are contraceptive pills at preventing HIV?  **(Show card)** | Very effective 1  Somewhat effective 2  Not too effective 3  Not at all effective 4 |  |
| 503 | How effective are partial penetration (removing penis before ejaculation) at preventing HIV?  **(Show card)** | Very effective 1  Somewhat effective 2  Not too effective 3  Not at all effective 4 |  |

| SEXUAL BEHAVIORS - SELF-ADMINISTRATIONHOW TO FILL THE ANSWER:  - Circle the relevant code(s) or write the answer in the requested area: Other (specify): - Select only 1 answer for each question. For the questions with instruction **“You can circle many answers or multiple choices”**, you can select more than one options. - Please note the Skip colum on the right - If you select the wrong answer, please cross like this and select the right answer. |
| --- |

| SECTION 6: SEXUAL BEHAVIORS | | | |
| --- | --- | --- | --- |
| **QID** | **Questions** | **Answers** | **Skip** |
| 601 | Have you ever had sexual intercourse?  *For the purposes of this survey, sexual intercourse means that (1) the penis is in the vagina, or (2) penis is in anus, or (3) finger or oral sex between two women or between men and women)* | Yes 1  No 2  No response 9 | If No, skip to Q701 |
| 602 | How old were you when you **first** had sexual intercourse? | Age in years  Don’t know 88  No response 99 |  |
| 603 | With whom did you have first sexual intercourse? | Boyfriend 1  Fiance 2  Husband 3  Someone you knew 4  Stranger 5  Other (specify) 6 |  |
| 604 | When did your first sex occur? while you were living in your home or while you were living in the IZ? | While living rural areas 1  While living in the IZ 2  No response 9 |  |
| 605 | Did you and/or your partner use condoms during your first sex? | Yes 1  No 2 | If No, skip to Q607 |
| 606 | Who initiated condom use? | You 1  Your partner 2  Both 3 |  |
| 607 | **Skip to Q1709 if you have never married**  Have you had sex before marriage? | Yes 1  No 2  No response 9 | If No, skip to Q609 |
| 608 | With whom have you had sex before marriage?  **(Multiple choices)** | Boyfriend who are now husband 1  Other boyfriend 2  Other (specify) 6 |  |
| 609 | Have you had sexual intercourse in the last 6 months? | Yes 1  No 2  Refused 9 | If No or refused skip to Q614 |
| 610 | How many sexual partners have you had in the past 6 months? | Number of partners  Don’t know 88  Refused 99 |  |
| 611 | Among them who are they?  **(Multiple choices)** | Husband 1  Boyfriend 2  Casual partners 3 |  |
| 612 | In the last 6 months, how often did you use condoms with **your regular sexual partners** (husbands/lovers)? | None 0  Always 1  Often 2  Some times 3  Rarely 4  Don’t remember 8  Refused 9 |  |
| 613 | In the last 6 months, how often did you use condoms with **your casual sexual partners**? | None 0  Always 1  Often 2  Some times 3  Rarely 4  Don’t remember 8  Refused 9 |  |
| 614 | When was the last time you have had sexual intercourse?  **(If more than 1 year, specify number of years. If less than 1 year, specify number of month(s). If less than 1 month, specify number of day(s))** | days ago  months ago  years ago |  |
| 615 | What is/was your relationship to the person you had sex with the last time? | Husband 1  Fiance 2  Lover (boyfriend) 3  Casual partner 4  Other (specify) 6 |  |
| 616 | Did you and your partner use condom during the last sex? | Yes 1  No 2 | If No, skip to 618 |
| 617 | Who initiated condom use? | You 1  Your partner 2  Both 3 |  |
| 618 | **Skip to Q619 if you have never married**  Have you ever had extra-marital sex? | Yes 1  No 2  Refused 9 |  |
| 619 | Have you ever engaged in sex in exchange for money? | Yes 1  No 2  Refused 9 |  |
| 620 | Which of these statements are true for you? | I have sex only with men 1  I have sex with both men and women 2  I have sex only with women 3  Refused 9 |  |
| 621 | Have you ever drunk alcohol either before or during sex? | Yes 1  No 2 |  |

| SECTION 7: USE OF HEALTH SERVICES Now I am going to ask you some questions about your use of health services | | | |
| --- | --- | --- | --- |
| **QID** | **Question** | **Answers** | **Skip** |
| 701 | Since you have been working in the IZ, have you used SRH/HIV services? | Yes 1  No 2 | If 2, skip to Q705 |
| 702 | What SRH or HIV services did you use?  **(Multiple choices)** | Contraceptive methods 1  Pregnancy care and delivery..... 2  Miscarriage/Post-abortion care  services 3  HIV test..... 4  STI treatment..... 5  Gynaecological exam.......... 6  Care for gender based violence 7  Counselling SRH 8  Information on SRH 9  Other (specify)____________ 96  Don’t know/don’t remember 99 |  |
| 703 | Where did you use the services?  **(Multiple choices)** | Clinic in the IZ 1  Private clinics 2  Private hospitals 3  Commune health center 4  District Health Center 5  Public hospital (district/provincial/ central) 6  Family planning association 7  Pharmacy 8  Others (specify) 9 |  |
| 704 | What are your general feelings about the SRH/HIV services you have accessed? | Very good 1  Good 2  Neutral 3  Not good 4 |  |
| 705 | What barriers exist to accessing HIV/SRH services when you need them?  **(Multiple choices)** | Timing is not convenient 1  Quality of services is not good 2  Staff negative attitude 3  Expensive 4  Discrimination 5  Lack of confidentiality 6  Inconvenience place/Too far to go 7  Others (specify) 8 |  |
| 706 | Since you have been working in the IZ, have you used other health services beside SRH/HIV services? | Yes 1  No 2 | If 2, stop here |
| 707 | What kind of services did you use?  **(Multiple choices)** | Some common health problems 1  Vaccination..... 2  Routine check – up 3  To get mental health and psychosocial support 4  Other (specify)________________ 6 |  |
| 708 | If yes, where did you use the services | Clinic in the IZ 1  Private clinics 2  Private hospitals 3  Commune health center 4  District Health Center 5  Public hospital (district/provincial/ central) 6  Family planning association 7  Pharmacy 8  Others (specify) ______________ 96 |  |

|  |  |  |  |
| --- | --- | --- | --- |

**THANK YOU!**
